# History of a Case in Which Symptoms of Pulmonary Consumption Were Suddenly Relieved by the Expectoration of a Piece of Carious Bone

**Published:** 1786

**Authors:** Charles Holman

**Affiliations:** Surgeon at Milverton, in Somersetshire


III. *History of a Case in which Symptoms of pulmonary Consumption were suddenly relieved by the Expectoration of a Piece of carious Bone. By Mr.* Charles Holman, *Surgeon, at Milverton, in Somersetshire.*

JOHN Dyte, of the parish of Milverton, aged forty-two years, applied to me on the 26th of April, 1785, for the cure of a cough, attended with an hæmoptoe at irregular intervals, and a considerable expectoration of ill-favoured pus. His pulse was small and quick; he complained of pain in his left side, near the fourth rib, (reckoning from above downwards;) and had for some time been subject to a colliquative diarrhœa, and to profuse night sweats, by which he was so much reduced as to be seemingly in the last stage of a pulmonary consumption.

Before I saw him he had taken a variety of remedies, and had occasionally lost a few ounces of blood; but from none of them had he experienced any good effect, except from the blood-letting, which, he observed, had generally afforded him a little temporary relief.

In the apparently hopeless state in which I found him, my view was merely to palliate his complaints; complaints; and, for this purpofe, I recommended a proper dofe of thebaic tincture at bed-time, to mitigate his cough, and gave him fome directions with refpect to regimen. He readily agreed to adopt any plan I fhould lay down for him; but before many days had elapfed, a fudden and unexpected event took place, which laid the foundation of his recovery. This happened on the 3d of May. His cough had come on the evening before with greater violence than ufual, and about midnight he coughed up a pint of blood, but without finding his cough at all diminifhed by this difcharge; on the contrary, it continued with increafed violence till about two o'clock in the morning, when he felt a folid body come from his throat (as he termed it) into his mouth. I faw him in the courfe of the day. His unfavourable fymptoms had fuddenly ceafed, and he had then only a flight cough remaining; fo that he was now in high fpirits, and confident of his recovery.

On examining the fubftance he had coughed up, I found it to be a piece of bone in a very carious ftate, weighing fix grains, and meafuring feven-eighths of an inch in circumference, and fix-eighths of an inch in length.

On

On queſtioning him with reſpect to his recollection of the lodgement of any ſuch ſubſtance, he told me that about fifteen years ago he remembered to have felt a bone lodge in the upper part of his throat while he was at breakfaſt, ſo as wholly to prevent deglutition. Surgical aid was called for, and a probang inſtantly removed the difficulty of ſwallowing; but from that period he became ſubject to a cough, which gradually brought on the ſymptoms of phthiſis already enumerated, and continued till after the expectoration of the bone in the manner I have related. In ſhort, a month after this happened, he was able to earn his bread as a day labourer, and is now in good health.

The eaſe with which deglutition was performed immediately after the uſe of the probang, and the cough which immediately ſucceeded, are both ſtrong reaſons for inducing us to ſuppoſe that the bone in this caſe was forced into the trachea; but the great ſenſibility of that canal, and the dreadful ſymptoms which are excited even by the falling of a drop of fluid into it, are great arguments againſt ſuch an opinion. — It might reaſonably be ſuppoſed that ſo large an extraneous ſubſtance would have occaſioned inſtant ſtrangulation, or that

ſuch

ſuch a degree of inflammation would have enſued as muſt have proved fatal.— Suppoſing it to have been forced into the looſe cellular membrane before the epiglottis, or between the oeſophagus and trachea, juſt at their commencement, it would in either ſituation, from its vicinity, have proved a conſtant ſtimulus to the bronchial veſſels, and been productive of more or leſs of pulmonic affection; but in that caſe it could hardly, I think, have afforded the inſtantaneous relief which the patient recollects to have felt in deglutition: but whatever might be the ſeat of the bone, the event of the caſe will ſerve (with a variety of others) to ſhow the great degree of violence which the human frame, under certain circumſtances, is capable of ſupporting.

*Milverton,*

January 11, 1786.

